# Role of the Bed Nucleus of the Stria Terminalis in PTSD: Insights From Preclinical Models

**DOI:** 10.3389/fnbeh.2019.00068

**Published:** 2019-04-05

**Authors:** Olivia W. Miles, Stephen Maren

**Affiliations:** Department of Psychological and Brain Sciences and Institute for Neuroscience, Texas A&M University, College Station, TX, United States

**Keywords:** PTSD, BNST, stress, fear conditioning, extinction, animal models

## Abstract

Post-traumatic stress disorder (PTSD) afflicts approximately 8% of the United States population and represents a significant public health burden, but the underlying neural mechanisms of this and other anxiety- and stressor-related disorders are largely unknown. Within the last few decades, several preclinical models of PSTD have been developed to help elucidate the mechanisms underlying dysregulated fear states. One brain area that has emerged as a critical mediator of stress-related behavioral processing in both clinical and laboratory settings is the bed nucleus of the stria terminalis (BNST). The BNST is interconnected with essential emotional processing regions, including prefrontal cortex, hippocampus and amygdala. It is activated by stressor exposure and undergoes neurochemical and morphological alterations as a result of stressor exposure. Stress-related neuro-peptides including corticotropin-releasing factor (CRF) and pituitary adenylate cyclase activating peptide (PACAP) are also abundant in the BNST, further implicating an involvement of BNST in stress responses. Behaviorally, the BNST is critical for acquisition and expression of fear and is well positioned to regulate fear relapse after periods of extinction. Here, we consider the role of the BNST in stress and memory processes in the context of preclinical models of PTSD.

## Introduction

Stress-related disorders, such as post-traumatic stress disorder (PTSD), are among the most debilitating neuropsychopathologies in the world. PTSD is nonexclusive and can affect individuals either directly or indirectly exposed to actual or perceived life-threatening events (Herman, 1992; Nievergelt et al., 2018). According to the National Institute of Mental Health (NIMH), an estimated 31% of United States adults experience symptoms of an anxiety disorder at some point in their lives (Kessler et al., 1995, 2004, 2005, 2009; Merikangas et al., 2010), and approximately 8% of Americans have PTSD at any given time (PTSD Statistics, 2018). With increasing costs associated with diagnosis, treatment (and sometimes even misdiagnosis and under treatment), lost work productivity, and high comorbidity rates with addiction disorders, anxiety-related disorders cost the US as much as $42.3 billion annually (PTSD Statistics, 2018).

Despite the continued economic and social burden of stress-related disorders, the molecular mechanisms underlying PTSD and other stress- and anxiety-related disorders are just now being thoroughly investigated (Insel et al., 2010; Cuthbert and Insel, 2013; Cuthbert, 2014; Insel, 2014), although common symptoms of these debilitating conditions are well known and include conditioned fear responses (Maren and Quirk, 2004; Todd et al., 2014; Giustino and Maren, 2015; Maren and Holmes, 2016; Maren, 2017; Trask et al., 2017; Ressler and Maren, 2019; Trask and Bouton, 2018). Once acquired, fear memories may span years or even decades in humans and contribute to the maintenance of fear and anxiety disorders like PTSD. Hence, there has been a considerable effort in the past several years to understand the neural mechanisms underlying acquisition of fear responses in animal models (Goode and Maren, 2017), particularly in regions associated with behavioral responses to stressor exposure. However, developing pre-clinical animal behavioral models to research the causes of anxiety disorders in the laboratory is challenging. Often, rodents are evaluated based on whether their behavioral changes following stressor exposure match a PTSD phenotype in humans after trauma (despite differences in motivation, emotion, and cognition between rodents and humans; for review, see Lezak et al., 2017), and whether these changes can be attenuated by medications used to treat stress-related disorders. Using this approach, early studies identified several brain regions, including the bed nucleus of the stria terminalis (BNST) and the amygdala, that are highly involved in mediating PTSD-like behaviors in rodents. Although these regions are in close proximity anatomically, they each make unique contributions to PTSD-associated behavioral phenotypes. Hence, we begin this review by discussing the differences between amygdala and BNST as they relate to PTSD. In subsequent sections, we address the role of the BNST in the stress response and identify neuropeptides that drive fear behavior. Finally, we consider current animal models of PTSD, critically review the specific involvement of BNST in each, and discuss the neural mechanisms driving behavioral manifestations of fear-related pathologies like PTSD. Based on these ideas, and a growing literature in BNST research, we suggest a critical role of BNST in PTSD-related behaviors and recommend continued research to explain how neural circuits involved in stress processing become dysregulated, and stay dysregulated, in response to trauma.

### Differentiating the BNST and Amygdala

#### Anatomical Distinctions

The BNST is a limbic system structure widely implicated in mediating behavioral responses to anxiety and stressor exposure (Walker et al., 2003; Choi et al., 2007; Hammack et al., 2009; Kocho-Schellenberg et al., 2014; Lezak et al., 2014a,b; Miles et al., 2018). It has been linked to anxiety and stress in both clinical and laboratory settings (Walker et al., 2003; Avery et al., 2016), and is subject to long-term physiological alterations after stressor exposure (Dumont et al., 2008) that enhance BNST function and mediate stress-related behaviors. The rodent BNST lies just dorsal to the neighboring amygdala and shares similar morphology. Although often grouped together as the “extended amygdala,” this term can be misleading, as studies have uncovered unique roles for each region in behavior that depend on their different projections, neuropeptides, and functional output. While a discussion of the multiple sub-regions and cell types associated with the BNST and amygdala are beyond the scope of this review, it is important to note the heterogeneity throughout each structure, as different regions and cell types may be activated in response to one stressor, for example, but not another (see Lebow and Chen, 2016). In short, BNST is often divided into anterior and posterior divisions that can be further categorized into distinct sub-regions including the well-recognized anterolateral, oval, and dorsomedial regions, among others. A heavy presence of androgen receptors, GABA receptors, adrenergic receptors, vesicular glutamate transporter 3 (VGLUT3) and PAC1 receptors are found in the anterior BNST, while posterior BNST also includes glutamate receptors and kainate receptors. Neuropeptide density also differs regionally: corticotropin releasing factor (CRF), dynorphin, enkephalin, pituitary adenylate cyclase activating peptide (PACAP), somatostatin, and neuropeptide Y (NPY) are all highly expressed in regions of the anterior BNST; glutamate, glutamate decarboxylase, and cholecystokinin, in contrast, have higher densities in the posterior BNST (see Lebow and Chen, 2016). Efferent projections both within the BNST and to surrounding regions [including amygdala, hypothalamus, ventral tegmental area (VTA), and lateral septum] help relay emotion-related information, whereas afferent projections [from frontal cortex, locus coeruleus (LC), ventral subiculum, VTA, amygdala, and even olfactory bulb] help integrate information about emotion.

The anatomy of the amygdala has also been reviewed elsewhere, but it includes the basolateral amygdala (which further subdivides into lateral and basal divisions, appropriately), the central amygdala (CeA; subdivided into lateral and medial components), and medial amygdala (Eleftheriou, 2013). Neuronal diversity in the amygdala is similar to the BNST: BLA contains glutamatergic neurons that serve to integrate and relay information between prefrontal cortex and hippocampus, among other regions, as well as GABAergic interneurons that gate information flow within the amygdala (Ramikie and Patel, 2012). The CeA, in turn, contains GABAergic neurons that integrate excitatory information from surrounding regions and project to the hypothalamus and BNST as well as downstream targets in the brainstem. Hence, the BLA serves as a sensory interface to the amygdala that directs the CeA to coordinate behavioral responses to biologically significant events.

#### BNST vs. Amygdala in Behavior Regulation

Although the BNST and amygdala are neuroanatomically distinct, they often communicate through CRF-containing projections: the BNST transmits information about stress-state through CRF projections to the amygdala, which then gets transmitted to hypothalamic-pituitary-adrenal (HPA) axis (Walker et al., 2003) neurons to stimulate the stress response system. Stress responses are generally protective short-term (Selye, 1955; Gold, 2015), but chronic stressor exposure results in many deleterious physiological and neuropsychological changes including increased risk for psychiatric disease (Vale, 2005; Pittenger and Duman, 2008; Gold, 2015). Upon stressor exposure, several stress response systems are activated, including the HPA axis and the sympathetic nervous system (SNS), as well as neural circuits important for emotional behavior such as fear and anxiety. Brain areas involved in regulating these physiological and emotional processes, such as the BNST and amygdala, undergo neurochemical and morphological changes following stressor exposure; these changes may underlie several mental health disorders (including PTSD and depression, among others; Hammack et al., 2010, 2012; Maren and Holmes, 2016).

An effort to delineate the behavioral responses dependent on BNST activity has been ongoing since Davis and colleagues initially argued that the BNST mediates a delayed response system following stressor exposure (in which the stress response system becomes activated sometime after stressor exposure rather than in direct response to a footshock, for example—akin, perhaps, to human states of anxiety; Walker et al., 2003; Davis et al., 2010). Notably, these early studies showed that lesions to CeA attenuated conditioned fear responses, but did not affect anxiety-like responses; in contrast, BNST lesions lessened anxiety-like responses, but not fear responses (Walker and Davis, 1997; Walker et al., 2003), illustrating an important distinction between the BNST and amygdala. Since then, several additional studies have suggested a purely temporal role of the BNST in fear learning (Davis et al., 2010; Hammack et al., 2015; Goode et al., 2018) although the BNST also acts in response to contextual cue reminders of perceived (i.e., sustained; *not* immediate) threat. Amygdala activation, however, seems critical to the immediate threat. Human and animal studies corroborate this idea (Hammack et al., 2010; Alvarez et al., 2015; Fox et al., 2015). A functional magnetic resonance imaging (fMRI) study of humans with phobias found increased BNST (and *not* amygdala) activation in response to an image of phobia-related objects (Straube et al., 2007), but dual activation of BNST and amygdala in response to current phobia-related challenges. In rodents, local infusion of AMPA-receptor antagonist NBQX into BNST decreased fear-potentiated startle but had no effect when infused into amygdala, indicating a selective mediation of sustained fear responses by the former (for review, see Davis et al., 2010). Hence, BNST appears to mediate trepidation while amygdala comes on board only in the threat of imminent danger (Goode and Maren, 2017).

#### BNST vs. Amygdala in Pavlovian Fear Conditioning

The general distinction between BNST and amygdala has been carried over to the fear-conditioning realm. Both structures have been widely implicated in the dysregulated stress response observed in psychiatric patients, but the BNST, in particular, has provided insight on fear memory neural circuitry that may underlie PTSD. Indeed, Pavlovian fear conditioning, where laboratory animals associate an aversive event (the unconditioned stimulus, or US; i.e., a footshock) with a cue that predicts the event (the conditioned stimulus, or CS; i.e., a tone), is believed to mediate fear learning associated with PTSD. The acquisition, expression, extinction and relapse of fear memories is perhaps the most common symptom of PTSD and often manifests as behavioral responses to trauma-related cues. Studying the learning mechanisms associated with fear memories is critical to understanding the neural circuitry behind the development, maintenance, and subsequent treatment of PTSD. However, BNST circuits become activated in response to limited stimulus modalities. The status of a CS as a contextual stimulus does not appear to rely on BNST activity (although Zimmerman and Maren demonstrated that BNST inactivation blocks freezing to a conditioning context, but not a CS, suggesting that BNST activation plays a role in the expression of contextual fear; Zimmerman and Maren, 2011; Hammack et al., 2015). In contrast, long-duration stimuli (and not short) does involve BNST (Waddell et al., 2006; Hammack et al., 2015). Furthermore, BNST activation does not appear to become more or less prevalent depending on how long a stressor may be presented (Walker et al., 2009; Hammack et al., 2015). BNST circuits may also process ambiguous threat signals: BNST inactivation reversed freezing to a backward CS (ambiguous signal for shock onset that occurs after shock) but did not affect freezing in response to CS’s that preceded shock exposure (Goode et al., 2018). Research on stress-network contributions has further informed this debate: ultra-high-field magnetic resonance imaging studies observed CeA and BNST responses to sustained threat and demonstrated decreases in intrinsic functional connectivity to regions including ventral medial PFC (Torrisi et al., 2018). Indeed, the role of vmPFC in human emotion regulation has been well studied (Dickie et al., 2008; Rougemont-Bücking et al., 2011; Bisson et al., 2013; Garfinkel et al., 2014), and if vmPFC drives BNST activity, activation of behavioral and physiological components of PTSD-like symptoms may occur. Further research should explore the direct connections between BNST, amygdala, and surrounding regions to identify involvement in the multiple stages of Pavlovian fear conditioning. Understanding these connections and how dysregulated neural circuitry drives trauma-related behaviors will become critical in developing new therapies.

## BNST Neuropeptides Involved in PTSD

The heterogeneity of the BNST, and the neuropeptides present within its nuclei suggests that the regulation of BNST activity is complex. The BNST is thought to aid communication between limbic system structures and emotional processing systems, insofar as it projects to and receives projections from areas responsible for both emotional and physiological responses to a stressor. As such, the BNST may have a prominent role in mediating stress disorders (i.e., PTSD; Hammack et al., 2010; Roman et al., 2014; Hammack and May, 2015). Furthermore, the BNST may not only be activated by stressor exposure but may undergo physical alterations as a result of stressor exposure (Lezak et al., 2014a); BNST neurochemistry and morphology is altered with repeated exposure to stress. In a study of unpredictable stress in rats, several researchers observed an increase in BNST volume and behavioral manifestations of stress after 28 days of chronic stressor exposure (McEwen and Chattarji, 2007). Hence, the BNST appears well suited to mediate stress-related behavior.

### Corticotropin Releasing Factor

CRF is widely regarded as a key regulator of the stress responses system (Bale and Vale, 2004; Slominski et al., 2013) as CRF and its cognate receptors, CRFR1 and CRFR2, are abundant in stress-related brain regions including BNST. CRF also initiates HPA axis activation by binding to CRFR1 receptors in the anterior pituitary after stressor exposure (Tsigos and Chrousos, 2002; Bale and Vale, 2004), which suggests CRF activation may mediate behavioral and physiological stress responses related to PTSD. The BNST oval nucleus, in addition to containing its own CRF, also receives CRF inputs from the CeA, BLA, parabrachial nucleus (PBn), hippocampus, and medial prefrontal cortex (mPFC), and contains CRF afferents to regions of the hypothalamus (i.e., paraventricular nucleus), limbic system structures and brainstem nuclei that mediate emotional behavior (Crestani et al., 2013).

Perhaps not surprisingly, CRF receptor expression is correlated with stress- and PTSD-related behavior: CRFR1 expression increases after stressor exposure in BNST, while CRFR2 expression decreases in certain sub-regions of BNST (Elharrar et al., 2013). As CRFR1 antagonists can block the effects of stressor exposure, it is thought that stress effects are mediated by CRFR1 (Binder and Nemeroff, 2010). In support of this, CRFR1 knockout mice exhibit decreased anxiety-like behavior (Timpl et al., 1998). However, CRFR2 receptors appear to mediate sustained fear and actually attenuate stress responses after the threat has passed (Bale and Vale, 2004). Mice with PTSD-like symptoms also show increased levels of BNST CRFR2 mRNA levels, and lentiviral knockdown of CRFR2 attenuates anxiety-like behavior (Lebow et al., 2012). Indeed, CRFR2 activation may recruit “coping genes” and reduce PTSD symptoms (Lebow and Chen, 2016). Hence, an important role of CRF receptors has been implicated in stress- and anxiety-related behaviors. In the clinical realm, it is interesting to note that CRF antagonists have been developed to treat stress-related illnesses, but have thus far been unsuccessful in treating behavioral disparities related to the disorder (Zorrilla and Koob, 2010).

Research on behavioral stress responses, such as contextual fear conditioning, has also been shown to increase CRF mRNA in dorsal regions of the BNST (Shalev et al., 2001; Davis et al., 2010). Recently, Pomrenze et al. (2019) showed a distinct CRF-mediated CeA to BNST circuit related to the generation of anxiety-like behaviors in rodents. Stress has also been shown to increase Fos expression (indicative of neuronal activity; Lin et al., 2018) that can be reduced by CNO activation of Gi-coupled DREADDs in PBN regulating BNST CRF neurons (Fetterly et al., 2019), further implicating BNST CRF neurons in response to stressor exposure. Optogenetic activation of CRFR2 neurons has also been shown to decrease anxiety and reduce PTSD-like symptoms in rodents (Henckens et al., 2017), suggesting the importance of CRF receptors in modulating behavioral outputs associated with stressor exposure. Furthermore, lateral hypothalamus-BNST circuits mediating emotional states, such as PTSD-related anxiety, depends on CRF activation (Giardino et al., 2018). Several researchers have observed increases in CRF following chronic variable stress paradigms in the dorsal lateral aspect of BNST—changes that are linked to an increase in anxiety-like behavior (Lee and Davis, 1997; Schulkin et al., 1998) and subsequent PTSD-like behaviors. Additionally, the amygdala is a major source of CRF afferents to the LC that modulate noradrenergic (NE) activity, and NE afferents directly innervate CRF-containing amygdala neurons (Kravets et al., 2015). Indeed, hyperarousal is mediated by NE BNST projections (Forray and Gysling, 2004), and is a common symptom of PTSD in humans. Hence, if CRF is downstream of stress-activated norepinephrine, integration may occur in the BNST. In support of these findings, patients exhibiting symptoms of PTSD in a clinical setting (Arató et al., 1989; Nemeroff et al., 1991; Baker et al., 1999; Ressler et al., 2011) have higher levels of CRF in their system compared to healthy controls. Depressed individuals also have heightened levels of CRF mRNA in BNST and amygdala (Merali et al., 2006) which, given the high levels of comorbidity between PTSD and depression (Kessler et al., 1995; Flory and Yehuda, 2015), further implicates CRF as a critical component of PTSD symptomology. Notably, using animal models, Dunn and Berridge (1987) showed that central CRF administration produces the same physiological effects as those associated with stressor exposure: increased heart rate, increased anhedonia and anorexic-like behaviors (Dunn and File, 1987), low sex drive (Dunn and Berridge, 1990), and reduced social interaction (Dunn and File, 1987). Hence, CRF activity regulates stress- and PTSD-related behavior.

### Pituitary Adenylate Cyclase Activating Peptide

PACAP has recently been implicated as a key regulator of peptide signaling in stress-related brain regions (Stroth et al., 2011; Hammack and May, 2015), and patients with PTSD show dysregulated PACAP activity that correlates with the severity of the disorder (Hashimoto et al., 2011, 2016; Ressler et al., 2011). A neurotrophic factor, PACAP promotes cell survival of multiple neuron types including progenitor cells, dorsal root ganglion cells, cerebellar granule cells, and peripheral sympathetic neurons (Stroth et al., 2011). Importantly, PACAP has been shown to increase cell survival in response to stressor exposure (Stroth et al., 2013), and regulates CRF *via* upstream activation in several stress-related regions to modulate CRF release and subsequent HPA axis activation (Gray and Cline, 2019). PACAP and its cognate G-protein coupled receptor, PAC1, are highly expressed in areas that project to the HPA axis, including the PVN where PACAP is heavily co-localized with CRF neurons (Hannibal et al., 1995). Furthermore, CRF transcription is increased in response to PACAP in hypothalamic cells (Stroth et al., 2011). Intracerebroventricular administration of PACAP has also increased CRF mRNA in PVN (Hashimoto et al., 2010), and PACAP-immunoreactive fibers form synapses in close proximity to CRF-expressing neurons in the PVN and BNST (Sherwood et al., 2000; Missig et al., 2014, 2017; Roman et al., 2014) suggesting PACAP may regulate neuroendocrine and behavioral responses to stressor exposure *via* CRF-dependent mechanisms. Indeed, PACAP null mice exhibit long-term HPA axis activation in response to chronic stressors, a finding that supports PACAP’s role in regulating the HPA axis during stressor exposure (Vaudry et al., 2005; Hammack et al., 2010). PACAP-deficient mice also exhibit lower CRF peptide content in PVN and subsequently demonstrate attenuated stress-hormone release after stressor exposure (Stroth and Eiden, 2010), indicating upstream activation of CRF activity by PACAP neurons. Hence, PACAP is involved in the regulation of PVN CRF neurons that activate HPA axis activity and subsequent stress response systems.

Recently, researchers observed that intra-BNST infusion of PACAP mimics behavioral and physiological responses to stressor exposure (Hammack et al., 2009; Miles et al., 2018). For example, when exposed to a 7-day chronic variable stress paradigm, rats exhibit anxiety- and stress-like behaviors that correspond with a significant increase in PACAP and PAC1 receptors in the BNST. Bilateral intra-BNST infusion of PACAP on its own is also sufficient to cause these same stress-related behaviors (Kocho-Schellenberg et al., 2014; Lezak et al., 2014a,b; Roman et al., 2014). Indeed, PACAP infusions mimic chronic stress-related responses by increasing startle and anxiety-like behavior on the elevated plus maze (i.e., rodents spend more time in the closed arm, indicative of anxiety), and elevating circulating corticosterone levels (Hammack et al., 2009; Stroth et al., 2011; Kocho-Schellenberg et al., 2014; Lezak et al., 2014a,b; Roman et al., 2014; King et al., 2017b). Additionally, the bilateral intra-BNST infusion of a PAC1 receptor antagonist reduced stress-induced consequences of repeated variable stress, suggesting that BNST PACAP is necessary for the stress responses observed (Roman et al., 2014). Combined, these data implicate PACAP as an important regulator of stress-related pathologies (Hammack et al., 2012) including PTSD.

### Neuropeptide Y

Translational research also points to the NPY system as a critical mediator of stress responses. Although NPY is expressed widely throughout the brain, mRNA and peptide content have been observed specifically in the BNST. Interestingly, the heightened expression in this region may be due to axons from NPY interneurons synapsing in BNST rather than local expression among BNST neurons themselves (Kash et al., 2015), although NPY receptors also densely populate the BNST, which lends support to its label as an “anti-stress” peptide. Indeed, NPY exhibits anxiolytic properties (Sajdyk et al., 2004; Reichmann and Holzer, 2016), perhaps due to its heightened presence between the hypothalamic arcuate nucleus (a major source of NPY), the PVN (the major source of CRF; Reichmann and Holzer, 2016), and the BNST. NPY also innervates the BNST and facilitates afferent cellular and circuit-based activity within limbic system structures including the amygdala.

Few studies have observed the direct effects of BNST NPY manipulations and behavioral output as it relates to PTSD, although chronic restraint stress (discussed in detail below) has been shown to increase NPY expression in BNST in mice susceptible to stress-effects (DBA/2J mice; Pleil et al., 2012, 2015), suggesting an involvement in NPY activity after stressor exposure. Appropriately, rodents exposed to chronic variable stress also show reduced NPY levels in BNST and amygdala (Kautz et al., 2017). Intranasal administration of NPY prior to Single Prolonged Stress (SPS) attenuated PTSD-like behavior in rats (Serova et al., 2013) and reversed PTSD-like behavior when administered after stressor exposure (Serova et al., 2014). Human patients with PTSD exhibit consistent alterations in peripheral levels of NPY (Rasmusson et al., 2000) and combat veterans with a diagnosis of PTSD had significantly lower levels of cerebrospinal fluid NPY than combat-experienced controls who did not develop PTSD-like symptoms (Sah et al., 2014). Hence, NPY involvement has been implicated in stress- and PTSD-related behaviors (Pleil et al., 2015), but more research is needed to determine BNSTs role in this activity.

### Cortisol/Corticosterone

A steroid hormone produced by the adrenal glands, cortisol (in humans; corticosterone in rodents) is known to be heavily involved in stress response systems. Although cortisol travels through the blood stream, receptors for cortisol are present on almost every bodily cell, facilitating vastly different effects depending on the location of peptide-receptor binding. The secretion of cortisol is controlled mainly by activation of the HPA axis through PVN CRF action after stressor exposure. Notably, BNST is anatomically situated to appropriately integrate synaptic activity from limbic system structures monitoring negative valance and PVN, and lesions to BNST attenuate corticosterone response to stress-related contextual stimuli (Sullivan et al., 2004) indicating an important role of BNST in stress- and PTSD-related behaviors. Furthermore, corticosterone injections in rodents facilitate an increase in anxiety-like behavior coupled with a decrease in dorsolateral BNST activity (Conrad et al., 2011), suggesting a distinct role of BNST/corticosterone interactions in stress responding.

The development of PTSD and other stress-related disorders facilitates a change in cortisol levels in humans. Recent data indicates lower levels of cortisol could be used as a predictor of risk to develop PTSD (Steudte-Schmiedgen et al., 2015). In rodent models, BNST inactivation leads to increased systemic corticosterone following restraint stress (see below; Myers et al., 2014), although sub-nuclei of the BNST may have different roles in mediating corticosterone release (Lebow and Chen, 2016). Intra-BNST (but not intraventricular) PACAP infusion increased plasma corticosterone levels in males and females, suggesting that BNST PACAP plays a key role in regulating stress responses (Lezak et al., 2014a). However, stress-induced elevations in corticosterone may not drive BNST peptide expression, as corticosterone treatment does not increase BNST PACAP transcript levels (Lezak et al., 2014b). Interestingly, PACAP knockout animals show reduced corticosterone levels after emotional stress (Ressler et al., 2011), and BNST PAC1 receptor antagonism blocks corticosterone release in a sensitized stress model (Roman et al., 2014). Recently, researchers showed that emotional stressors (i.e., open-field exposure or restraint stress), but not physical stressors, attenuate corticosterone release in PACAP knockout mice (Tsukiyama et al., 2011). Furthermore, blunted basal corticosterone levels appear to serve as a risk factor for PTSD-like behaviors in rats (Danan et al., 2018) and intraperitoneal injection of corticosterone following fear memory reactivation reduces retrieval of strong contextual memories (Abrari et al., 2008). Finally, exposure to high levels of corticosterone leads to impaired fear extinction to contextual freezing (Gourley et al., 2009), which may be BNST dependent. Hence, hypocortisolism and subsequent HPA axis alterations may serve as a risk factor for PTSD development, although additional research is needed to thoroughly examine the relationship between cortisol release and BNST activity.

## Animal Models of PTSD

Several animal models of PTSD have been developed (recently reviewed in detail by Flandreau and Toth, 2018) to model different aspects of PTSD symptomology widely used to study neural and behavioral manifestations of trauma. The remainder of this review discusses the role of the BNST in each of these models.

### Restraint Stress

Restraint stress, in which a rodent is placed in an enclosed chamber and allowed minimal movement, is typically used to model PTSD-like anxiety symptoms in rodents and has been shown to induce structural remodeling throughout stress-related regions of the brain (Pham et al., 2003). In rats, restraint stress disrupts fear extinction compared to non-stressed animals (Izquierdo et al., 2006) consistent with human PTSD literature. Adami et al. (2017) showed that, after restraint, BNST glutamatergic neurotransmission in rodents influences changes in heart rate and tail skin temperature *via* co-activation of N-Methyl-D-aspartate (NMDA) and non-N-Methyl-D-aspartate (NMDA) receptors. Several studies have also demonstrated an involvement of CRF1 and CRF2 receptors (Oliveira et al., 2015), α1-adrenoceptors (Barretto-de-Souza et al., 2018), and endocannabinoid CB1 receptors (Gomes-de-Souza et al., 2016) in the BNST in cardiovascular adjustments during restraint stress (Oliveira et al., 2015). However, PVN CRF mRNA is not upregulated following restraint stress (Stroth et al., 2011) indicating upstream activation of CRF function (*via* PACAP, for example; Hammack et al., 2010; King et al., 2017b). Acute restraint also causes an increase in corticosterone release in mice, but this change is significantly attenuated in PACAP- and PAC1-deficient mice, suggesting that PACAP-PAC1 receptor binding in BNST may mediate central short-term effects of restraint stress (Mustafa et al., 2015). Relatedly, acute stress (i.e., a short, potent stressor) has been shown to elevate norepinephrine in the BNST: Schmidt et al. (2018) showed, using optogenetic-assisted fast-scan cyclic voltammetry, elevated norepinephrine release across several stimulation parameters and reduced sensitivity to norepinephrine auto-receptors in mice exposed to 5 days restraint stress. In contrast to reports of acute restraint, chronic immobilization increases BNST (but not amygdala) dendritic branching (Vyas et al., 2003), suggesting a role of stress in remodeling neurons in stress-related brain regions after the traumatic event that underlies PTSD. Chronic variable stress paradigms that include restraint stress (as well as pedestal stress, forced swim, footshock, and oscillation; but not single stress exposure) are also associated with increased levels of histone H2A-X phosphorylated at serine 139 (γH2AX), a marker of DNA damage associated with cell death in the BNST (Hare et al., 2018). Hence, chemical and morphological changes that occur in BNST as a result of restraint stress generate behavioral anxiety-like phenotypes that match human PTSD characteristics.

### Footshock Stress

One of the most widely accepted methods of stressor exposure, footshock stress has been used to study effects of a single traumatic stressor (modeling, appropriately, a single traumatic experience that leads to the development of PTSD) and fear conditioning (observing the mechanisms underlying stressor exposure; for review, see Goode and Maren, 2017; Flandreau and Toth, 2018; Goode et al., 2018). Fear conditioning using footshock stress generally includes tests for fear extinction, which is impaired in PTSD patients, and dependent on BNST activity. Interestingly, the presentation of footshock stress not only induces reliable freezing in response to previously neutral cues associated with the shock, but suppresses instrumental responding for food (conditioned suppression; Bouton, 1986; Waddell et al., 2006; Allcoat et al., 2015) and increases startle responses to non-related aversive stimuli (fear potentiated startle; Walker and Davis, 1997), both of which are mediated by BNST activity. Chronically stressed rats exposed to a single footshock stressor also show enhanced neuronal activation in regions associated with stress responses, including the BNST and BLA. Indeed, exposure to an aversive event, in general, has been shown to activate or modify BNST signaling (Daniel and Rainnie, 2016; Marcinkiewcz et al., 2016; Rainnie et al., 2017) but with very little uniformity.

GABAergic neurons are widespread within the rodent BNST and active during fear conditioning. Photoinhibition of BNST-VTA projections during footshock, for example, reduces freezing behavior in contexts previously associated with stressor exposure (Ch’ng et al., 2018) and decreases closed arm entries on an elevated plus maze (akin to anxiety-like behavior in PTSD patients; Jennings et al., 2013), indicating a potentially anxiolytic role of GABAergic pathways projecting to and from the BNST. Additionally, chronic exposure to unpredictable footshock stress *increases* serotonin release in the BNST and modifies cell type-specific distribution of serotonin receptors within the BNST and amygdala (Hazra et al., 2012). Appropriately, dysregulation of the GABAergic and serotonin systems increases anxiety-states and has been linked to the pathophysiology of PTSD (Krystal and Neumeister, 2009; Kelmendi et al., 2016).

Learned helplessness (LH) has also recently been classified as a potential model of PTSD (although was initially developed to model depression; Seligman, 1974; Maier and Seligman, 2016). Here, animals exposed to controllable or uncontrollable shock stress ultimately learn appropriate avoidance responses that may be BNST-dependent (Hammack et al., 2012). Notably, behavioral changes associated with controllable or uncontrollable shock presentation appear mediated by the controllability of the stressor rather than the shock stress itself (Hammack et al., 2012) and mimic anxiety-like states seen in PTSD and depression phenotypes. LH-associated shocks activate BNST neurons (Greenwood et al., 2005) that may drive CRF- or PACAP-induced behavioral responses. Given the high rates of comorbidity between depression and PTSD, continued research on the BNST mechanisms underlying LH may aid our understanding of the shared neural activity.

### Forced Swim

The forced swimming model of depression has long been used as a measure of antidepressant drug efficacy (although the translational implications of this model are arguably weak), and requires that rodents swim for 15 min in a deep water-filled tank. Typically, rodents demonstrate escape behaviors but eventually adopt an immobile posture when escape fails. Subsequent tests show the rodents becoming immobile earlier (generally accepted as a sign of depression), but are eventually re-mobilized with the administration of antidepressants (Pezuk et al., 2008). Because the immobility, or signs of “giving up,” mimics symptoms of mood disorders in humans, the forced swim test has been adopted as a model of PTSD. High levels of comorbidity between PTSD and depression suggest the two phenotypes share common neural circuitry underlying their respective behavioral manifestations.

BNST activity appears critical for both PTSD- and depression-like symptoms in rodents, and modulation of this region alters behavioral phenotypes associated with the disorders. BNST lesions (Pezuk et al., 2008) and temporary inactivation of synaptic transmission within the BNST (Crestani et al., 2010) result in increased immobility after multiple swim trials in both male and female rats compared to controls. Intraperitoneal injections of CRF antagonists (known to demonstrate antidepressant-like effects) decreased immobility (Jutkiewicz et al., 2005), as did the subcutaneous administration of δ opioid agonists (Broom et al., 2002), both of which may act on stress-related brain regions like BNST. CRF may also have a differential role at BNST and amygdala in forced swim: lentivirus overexpression of CRF in CeA attenuated swim-induced anxiety-like behaviors, but overexpression in BNST promoted depressive-like behaviors (Regev et al., 2011). Local BNST CRF administration also reduces activity in LC (an established brain region mediating arousal) following forced swim (Curtis et al., 1999), suggesting a role of stressors in mediating threshold activation of arousal. Furthermore, mice deficient in PACAP (upstream of CRF in BNST) show immediately increased immobility during forced swim (Hashimoto et al., 2009), indicating an importance of BNST CRF and PACAP in PTSD and depression phenotypes. Underwater holding has also been used to target PTSD phenotypes in rodents (Richter-Levin, 1998; Flandreau and Toth, 2018). Notably, 20–30 s of forced water submersion results in increased startle reactivity (Richter-Levin, 1998), which may be BNST-PACAP dependent (for review, see King et al., 2017a). The forced swim test of “behavioral despair” (Flandreau and Toth, 2018) has predictive validity for antidepressant medications (which have shown some efficacy in PTSD treatment; see Cryan and Kaupmann, 2005), but more research is needed to determine the relationship between models of depression, PTSD, and associated neural circuitry.

### Predator Based Psychosocial Stress

The predator-based psychological stress (PPS) model of PTSD is an ethologically relevant stressor based on a threat to survival and a lack of social support (both factors of PTSD in humans). Rodents exposed to 2,5-dihydro-2,4,5-trimethylthiazoline (TMT), a synthetic derivative of fox feces, exhibit intense stress responses mediated (in part) by the BSNT (Janitzky et al., 2015). Notably, the BNST receives afferent projections from the olfactory bulbs (Kang et al., 2009; Janitzky et al., 2015) and is well suited to integrate neural information, endocrine responses, and behavioral manifestations of stress. Indeed, temporary inactivation of BNST (but not amygdala) with muscimol (a GABAa receptor agonist) blocked TMT-induced freezing in rats (Fendt et al., 2003), supporting a role of BNST in unlearned fear. This activity may be regulated by interactions between CRF and GABAergic transmission within the BNST, as higher stress sensitivity in mice (measured by working memory tasks) could result from high CRF expression and low GABAergic signaling (Janitzky et al., 2014). Exposure to a cat reduces pCREB expression in the BNST (Blundell and Adamec, 2007), but increases pCREB in CeA (Adamec et al., 2006) of rats. Importantly, some models of predator exposure cause long-term (i.e., months-long) behavioral changes that mimic the lasting clinical symptoms of PTSD in humans.

### Social Defeat Stress

A particularly effective social stress model in rodents is the resident-intruder social defeat model in which mice are repeatedly exposed to an unfamiliar, dominant aggressor (Newman et al., 2018) and either socialize with this intruder (where the mice are then dubbed “resilient”) or become anti-social (and are considered more “susceptible” to trauma). This distinction is then used to study biological markers of trauma in the susceptible population. Social defeat stress mimics PTSD symptoms including anxiety- and depressive-like behavior in rodents that persist long after the initial stressor exposure; it is, therefore, high in translational validity and commonly used as a model of PTSD.

In mice, social defeat tests demonstrate increases in brain-derived neurotrophic factor (BDNF; critical for the growth, maturation, and survival of neurons) protein in the BNST of females (and not males; Greenberg et al., 2014) and exaggerated social withdrawal. Intraspecific confrontation between male and female rats, in which an aggressor socially defeats a subordinate, increased c-fos expression in BNST, showing a cellular change in stress-related regions specific to stressor exposure. Furthermore, selective antagonism of CRFR2 (but not CRFR1) receptors reduces defensive behavior following social defeat in Syrian hamsters when administered intracerebroventricularly or directly into BNST. Previous studies, however, demonstrate that CRF receptors modulate social defeat at the BNST (and not amygdala; Jasnow et al., 2004), suggesting that even ICV infusion may act specifically on BNST receptors (Cooper and Huhman, 2005). Despite altered activity of the BNST following social defeat, pCREB and pERK expression were unchanged (although perhaps these markers are not activated by social stimuli; Trainor et al., 2011). Finally, intermittent episodes of social stress escalate alcohol and cocaine intake in rats, but continuous exposure attenuates cocaine intake and increases alcohol intake (Newman et al., 2018), a behavioral change that may (like other models of stress-induced reinstatement to drug-seeking; see Miles et al., 2018) be BNST-dependent. Because BNST activity may drive PTSD-like symptomology, neurochemical and anatomical alterations after social defeat stress suggest a critical involvement of BNST in the maintenance and development of PTSD.

### Single Prolonged Stress (SPS)

This animal model is based on the finding that PTSD may be induced after a person experiences a single traumatic incident. While SPS causes a number of behavioral changes similar to those described in PTSD patients, little work has been done to determine the role of BNST activity in this model. However, exposure to 2-h restraint followed by forced swim and ether anesthesia does successfully reproduce neuroendocrine and behavioral characteristics of PTSD including HPA axis activation (Liberzon et al., 1997) and increased acoustic startle response (Khan and Liberzon, 2004). These responses, in turn, may be BNST dependent, as intra-BNST administration of AMPA antagonists block light-potentiated acoustic startle response (Walker and Davis, 1997). Furthermore, rats exposed to an SPS procedure showed reduced fear extinction learning (likely due to disrupted retention) that led to enhanced renewal (Knox et al., 2012). SPS also has been shown to decrease open arm exploration on an elevated plus maze (indicating an anxiogenic state; Qiu et al., 2016), which could be attributed to BNST activation (Butler et al., 2016). Given the overlap between SPS-induced behaviors and PTSD-related characteristics in humans, the BNST may be a key area of interest for future investigations in this model.

### Prenatal Stress

While not a model of PTSD *per se*, early life events often have long-lasting impacts on cardiovascular, neuroendocrine, and cognitive development (Harris and Seckl, 2011). Rodents that undergo restraint stress during pregnancy deliver offspring that demonstrate increased vulnerability to PTSD, anxiety disorders, and learning difficulties (Ward et al., 2000; Harris and Seckl, 2011). Early environmental challenges increase anxiety and depressive-like behavior as they age, corroborating human data (Meaney and Szyf, 2005). However, is it unclear whether HPA axis dysregulation and subsequent BNST activity, in these animals (and humans) is a cause or symptom of PTSD. Data from human women who were pregnant on September 11, 2001 and in/near the World Trade Center who developed PTSD show lower cortisol (Lupien et al., 2000) levels than women who did not develop PTSD (Yehuda et al., 2005). This reduction may be facilitated by BNST activation, as animal models of PTSD show lower levels of corticosterone following stressor exposure (see above). Offspring of these women also have significantly lower levels of cortisol compared to children from mothers without PTSD (Yehuda, 2002; Yehuda et al., 2005). Furthermore, extended amygdala CRF expression and receptor-bound activation were increased in rodent adult prenatally stressed offspring (Cratty et al., 1995; Ward et al., 2000). Elevated plasma corticosterone and adrenal hypertrophy were also observed in prenatally stressed rats (Ward et al., 2000), and prenatal stress leads to long-lasting increases in plasma corticosterone after restraint stress in adulthood (Louvart et al., 2009). CRF receptor antagonists can also attenuate the defensive withdrawal behavior of prenatally stressed offspring. Hence, prenatal stress may contribute to stress vulnerability later in life that manifests as susceptibility to stress-related disorders including PTSD.

### BNST-Related Sex Differences in PTSD

Adult women exposed to trauma demonstrate a two-fold higher lifetime prevalence of PTSD than their male counterparts (Kessler et al., 1995; Breslau et al., 1997; Olff, 2017), and although it is clear that psychosocial and biological factors may contribute, the underlying neural mechanisms of this discrepancy are not well understood. To date, an animal model of PTSD does not exist that reliably shows females developing and maintaining PTSD-like characteristics at a two-fold higher rate than males that would match clinical manifestations of PTSD (Shansky, 2015; but also see Pooley et al., 2018). While a thorough discussion of sex differences in trauma (including PTSD) is beyond the scope of this review, a few points regarding BNST sex differences relating to PTSD are worth noting.

In several learning conditioning paradigms thought to mimic PTSD development and maintenance, BNST activity enhances the effect of stressor exposure on males and not females (Bangasser et al., 2005, 2016; Bangasser and Wicks, 2017), suggesting that different circuits mediate stress-responses depending on sex. Indeed, the BNST itself is sexually dimorphic: the human BNST region is approximately 2.5 times greater in males than females, a sexual dimorphism that also appears in rodents (Allen and Gorski, 1990). This size difference corresponds appropriately with differences in neurochemical composition and connections to other sexually dimorphic nuclei (Simerly and Swanson, 1986) that mediate behavioral outputs associated with stressor exposure, including PTSD-like behaviors. The regulation of sex-related peptides may also be estrogen-dependent (Ramikie and Ressler, 2016). PACAP transcripts, for example, are increased in BNST in ovariectomized female rats after continuous exposure to estrogen (Ressler et al., 2011), and the PACAP system itself appears sexually dimorphic in stress-related regions (King et al., 2017a). CRF activation of the HPA axis is also stronger in females than males (Bangasser and Valentino, 2012, 2014), and CRF receptor activation in stress-related regions appears lower in females after acute stress (Bangasser et al., 2010). In a study of contextual fear conditioning, researchers found that intra-BNST infusion of allopregnanolone, a metabolite of progesterone, in males suppressed freezing behavior, but inhibiting allopregnanolone in females enhanced fear conditioning (Nagaya et al., 2015), suggesting a role of BNST in anxiety-related behaviors that depend on steroidal regulation. Sex-dependent differences in HPA axis activity (Stephens et al., 2016) also supports the divergence in stress-related behaviors observed in males compared to females. As mentioned, HPA axis dysregulation underlies stress-related disorders including PTSD and is linked to BNST activity. Continued research is needed to determine why females tend to show different neurochemical and behavioral responses to trauma than males and the underlying neural circuitry responsible for these differences.

## Conclusion

The BNST has long been implicated in stress- and anxiety-related behaviors, but has only recently been considered a potential therapeutic target for PTSD symptoms. A review of animal behavior in preclinical models of PTSD suggests that the BNST may underlie symptoms of stress-related disorders in humans. Indeed, the BNST appears critical for the acquisition and expression of fear and is well positioned to regulate fear relapse after periods of extinction that mimics fear relapse in PTSD patients. Stress-related peptides are also prevalent in this region, suggesting interactions between BNST and HPA axis activation mediates behavioral outputs. It is important to note, however, that BNST activity and subsequent peptide release may differ depending on the preclinical model under investigation. Furthermore, the BNST itself comprises multiple sub-regions, cell types, and peptide expression, all of which may not contribute equally to stress-related behavioral outputs. A more thorough understanding of how these different sub-regions contribute to PTSD-related symptomology will be important moving forward. Indeed, continued research in this area may aid the development of novel pharmacotherapeutic treatments for PTSD in humans.

## Author Contributions

OM and SM wrote and edited the manuscript.

## Conflict of Interest Statement

The authors declare that the research was conducted in the absence of any commercial or financial relationships that could be construed as a potential conflict of interest.

## References

[B1] Abrari K. Rashidy-Pour A. Semnanian S. Fathollahi Y. (2008). Administration of corticosterone after memory reactivation disrupts subsequent retrieval of a contextual conditioned fear memory: dependence upon training intensity. Neurobiol. Learn. Mem. 89, 178–184. 10.1016/j.nlm.2007.07.005 17702613

[B2] Adamec R. E. Blundell J. Burton P. (2006). Relationship of the predatory attack experience to neural plasticity, pCREB expression and neuroendocrine response. Neurosci. Biobehav. Rev. 30, 356–375. 10.1016/j.neubiorev.2005.04.004 16115684

[B3] Adami M. B. Barretto-de-Souza L. Duarte J. O. Almeida J. Crestani C. C. (2017). Both N-methyl-D-aspartate and non-N-methyl-D-aspartate glutamate receptors in the bed nucleus of the stria terminalis modulate the cardiovascular responses to acute restraint stress in rats. J. Psychopharmacol. 31, 674–681. 10.1177/0269881117691468 28198211

[B4] Allcoat D. Greville W. J. Newton P. M. Dymond S. (2015). Frozen with fear: conditioned suppression in a virtual reality model of human anxiety. Behav. Processes 118, 98–101. 10.1016/j.beproc.2015.06.011 26115568

[B5] Allen L. S. Gorski R. A. (1990). Sex difference in the bed nucleus of the stria terminalis of the human brain. J. Comp. Neurol. 302, 697–706. 10.1002/cne.903020402 1707064

[B6] Alvarez R. P. Kirlic N. Misaki M. Bodurka J. Rhudy J. L. Paulus M. P.. (2015). Increased anterior insula activity in anxious individuals is linked to diminished perceived control. Transl. Psychiatry 5:e591. 10.1038/tp.2015.84 26125154 PMC4490294

[B7] Arató M. Bánki C. M. Bissette G. Nemeroff C. B. (1989). Elevated CSF CRF in suicide victims. Biol. Psychiatry 25, 355–359. 10.1016/0006-3223(89)90183-2 2536563

[B8] Avery S. N. Clauss J. A. Blackford J. U. (2016). The human BNST: functional role in anxiety and addiction. Neuropsychopharmacology 41, 126–141. 10.1038/npp.2015.185 26105138 PMC4677124

[B9] Baker D. G. West S. A. Nicholson W. E. Ekhator N. N. Kasckow J. W. Hill K. K.. (1999). Serial CSF corticotropin-releasing hormone levels and adrenocortical activity in combat veterans with posttraumatic stress disorder. Am. J. Psychiatry 156, 585–588. 10.1176/ajp.156.4.585 10200738

[B10] Bale T. L. Vale W. W. (2004). CRF and CRF receptors: role in stress responsivity and other behaviors. Annu. Rev. Pharmacol. Toxicol. 44, 525–557. 10.1146/annurev.pharmtox.44.101802.121410 14744257

[B14] Bangasser D. A. Curtis A. Reyes B. A. S. Bethea T. T. Parastatidis I. Ischiropoulos H.. (2010). Sex differences in corticotropin-releasing factor receptor signaling and trafficking: potential role in female vulnerability to stress-related psychopathology. Mol. Psychiatry 15, 896–904. 10.1038/mp.2010.66 20548297 PMC2935505

[B15] Bangasser D. A. Santollo J. Shors T. J. (2005). The bed nucleus of the stria terminalis is critically involved in enhancing associative learning after stressful experience. Behav. Neurosci. 119, 1459–1466. 10.1037/0735-7044.119.6.1459 16420150 PMC3422871

[B11] Bangasser D. A. Valentino R. J. (2012). Sex differences in molecular and cellular substrates of stress. Cell. Mol. Neurobiol. 32, 709–723. 10.1007/s10571-012-9824-4 22488525 PMC3378920

[B12] Bangasser D. A. Valentino R. J. (2014). Sex differences in stress-related psychiatric disorders: neurobiological perspectives. Front. Neuroendocrinol. 35, 303–319. 10.1016/j.yfrne.2014.03.008 24726661 PMC4087049

[B13] Bangasser D. A. Wicks B. (2017). Sex-specific mechanisms for responding to stress. J. Neurosci. Res. 95, 75–82. 10.1002/jnr.23812 27870416 PMC5120612

[B16] Bangasser D. A. Wiersielis K. R. Khantsis S. (2016). Sex differences in the locus coeruleus-norepinephrine system and its regulation by stress. Brain Res. 1641, 177–188. 10.1016/j.brainres.2015.11.021 26607253 PMC4875880

[B17] Barretto-de-Souza L. Adami M. B. Benini R. Crestani C. C. (2018). Dual role of nitrergic neurotransmission in the bed nucleus of the stria terminalis in controlling cardiovascular responses to emotional stress in rats. Br. J. Pharmacol. 175, 3773–3783. 10.1111/bph.14447 30007000 PMC6135781

[B18] Binder E. B. Nemeroff C. B. (2010). The CRF system, stress, depression and anxiety-insights from human genetic studies. Mol. Psychiatry 15, 574–588. 10.1038/mp.2009.141 20010888 PMC3666571

[B19] Bisson J. I. Roberts N. P. Andrew M. Cooper R. Lewis C. (2013). Psychological therapies for chronic post-traumatic stress disorder (PTSD) in adults. Cochrane Database Syst. Rev. 12:CD003388. 10.1002/14651858.cd003388.pub4 24338345 PMC6991463

[B20] Blundell J. Adamec R. (2007). The NMDA receptor antagonist CPP blocks the effects of predator stress on pCREB in brain regions involved in fearful and anxious behavior. Brain Res. 1136, 59–76. 10.1016/j.brainres.2006.09.078 17239834

[B21] Bouton M. E. (1986). Slow reacquisition following the extinction of conditioned suppression. Learn. Motiv. 17, 1–15. 10.1016/0023-9690(86)90017-2

[B22] Breslau N. Davis G. C. Peterson E. L. Schultz L. (1997). Psychiatric sequelae of posttraumatic stress disorder in women. Arch. Gen. Psychiatry 54, 81–87. 10.1001/archpsyc.1997.01830130087016 9006404

[B23] Broom D. C. Jutkiewicz E. M. Folk J. E. Traynor J. R. Rice K. C. Woods J. H. (2002). Nonpeptidic delta-opioid receptor agonists reduce immobility in the forced swim assay in rats. Neuropsychopharmacology 26, 744–755. 10.1016/s0893-133x(01)00413-4 12007745

[B24] Butler R. K. Oliver E. M. Sharko A. C. Parilla-Carrero J. Kaigler K. F. Fadel J. R.. (2016). Activation of corticotropin releasing factor-containing neurons in the rat central amygdala and bed nucleus of the stria terminalis following exposure to two different anxiogenic stressors. Behav. Brain Res. 304, 92–101. 10.1016/j.bbr.2016.01.051 26821289 PMC4789084

[B25] Ch’ng S. Fu J. Brown R. M. McDougall S. J. Lawrence A. J. (2018). The intersection of stress and reward: BNST modulation of aversive and appetitive states. Prog. Neuropsychopharmacol. Biol. Psychiatry 87, 108–125. 10.1016/j.pnpbp.2018.01.005 29330137

[B26] Choi D. C. Furay A. R. Evanson N. K. Ostrander M. M. Ulrich-Lai Y. M. Herman J. P. (2007). Bed nucleus of the stria terminalis subregions differentially regulate hypothalamic-pituitary-adrenal axis activity: implications for the integration of limbic inputs. J. Neurosci. 27, 2025–2034. 10.1523/JNEUROSCI.4301-06.2007 17314298 PMC6673539

[B27] Conrad K. L. Louderback K. M. Gessner C. P. Winder D. G. (2011). Stress-induced alterations in anxiety-like behavior and adaptations in plasticity in the bed nucleus of the stria terminalis. Physiol. Behav. 104, 248–256. 10.1016/j.physbeh.2011.03.001 21396387 PMC3118978

[B28] Cooper M. A. Huhman K. L. (2005). Corticotropin-releasing factor type II (CRF-sub-2) receptors in the bed nucleus of the stria terminalis modulate conditioned defeat in Syrian hamsters *(Mesocricetus auratus)*. Behav. Neurosci. 119, 1042–1051. 10.1037/0735-7044.119.4.1042 16187832

[B29] Cratty M. S. Ward H. E. Johnson E. A. Azzaro A. J. Birkle D. L. (1995). Prenatal stress increases corticotropin-releasing factor (CRF) content and release in rat amygdala minces. Brain Res. 675, 297–302. 10.1016/0006-8993(95)00087-7 7796142

[B30] Crestani C. C. Alves F. H. F. Correa F. M. A. Guimarães F. S. Joca S. R. L. (2010). Acute reversible inactivation of the bed nucleus of stria terminalis induces antidepressant-like effect in the rat forced swimming test. Behav. Brain Funct. 6:30. 10.1186/1744-9081-6-30 20515458 PMC2887770

[B31] Crestani C. C. Alves F. H. Gomes F. V. Resstel L. B. Correa F. M. Herman J. P. (2013). Mechanisms in the bed nucleus of the stria terminalis involved in control of autonomic and neuroendocrine functions: a review. Curr. Neuropharmacol. 11, 141–159. 10.2174/1570159x11311020002 23997750 PMC3637669

[B32] Cryan J. F. Kaupmann K. (2005). Don’t worry ‘B’ happy!: a role for GABA(B) receptors in anxiety and depression. Trends Pharmacol. Sci. 26, 36–43. 10.1016/j.tips.2004.11.004 15629203

[B33] Curtis A. L. Pavcovich L. A. Valentino R. J. (1999). Long-term regulation of locus ceruleus sensitivity to corticotropin-releasing factor by swim stress. J. Pharmacol. Exp. Ther. 289, 1211–1219. 10336508

[B34] Cuthbert B. N. (2014). The RDoC framework: facilitating transition from ICD/DSM to dimensional approaches that integrate neuroscience and psychopathology. World Psychiatry 13, 28–35. 10.1002/wps.20087 24497240 PMC3918011

[B35] Cuthbert B. N. Insel T. R. (2013). Toward the future of psychiatric diagnosis: the seven pillars of RDoC. BMC Med. 11:126. 10.1186/1741-7015-11-126 23672542 PMC3653747

[B36] Danan D. Matar M. A. Kaplan Z. Zohar J. Cohen H. (2018). Blunted basal corticosterone pulsatility predicts post-exposure susceptibility to PTSD phenotype in rats. Psychoneuroendocrinology 87, 35–42. 10.1016/j.psyneuen.2017.09.023 29035710

[B37] Daniel S. E. Rainnie D. G. (2016). Stress modulation of opposing circuits in the bed nucleus of the stria terminalis. Neuropsychopharmacology 41, 103–125. 10.1038/npp.2015.178 26096838 PMC4677121

[B38] Davis M. Walker D. L. Miles L. Grillon C. (2010). Phasic vs sustained fear in rats and humans: role of the extended amygdala in fear vs anxiety. Neuropsychopharmacology 35, 105–135. 10.1038/npp.2009.109 19693004 PMC2795099

[B39] Dickie E. W. Brunet A. Akerib V. Armony J. L. (2008). An fMRI investigation of memory encoding in PTSD: influence of symptom severity. Neuropsychologia 46, 1522–1531. 10.1016/j.neuropsychologia.2008.01.007 18321537

[B40] Dumont E. C. Rycroft B. K. Maiz J. Williams J. T. (2008). Morphine produces circuit-specific neuroplasticity in the bed nucleus of the stria terminalis. Neuroscience 153, 232–239. 10.1016/j.neuroscience.2008.01.039 18343592 PMC3632387

[B41] Dunn A. J. Berridge C. W. (1987). Corticotropin-releasing factor administration elicits a stress-like activation of cerebral catecholaminergic systems. Pharmacol. Biochem. Behav. 27, 685–691. 10.1016/0091-3057(87)90195-x 2443934

[B42] Dunn A. J. Berridge C. W. (1990). Physiological and behavioral responses to corticotropin-releasing factor administration: is CRF a mediator of anxiety or stress responses? Brain Res. Rev. 15, 71–100. 10.1016/0165-0173(90)90012-d 1980834

[B43] Dunn A. J. File S. E. (1987). Corticotropin-releasing factor has an anxiogenic action in the social interaction test. Horm. Behav. 21, 193–202. 10.1016/0018-506x(87)90044-4 2886414

[B44] Eleftheriou B. (2013). The Neurobiology of the Amygdala: The Proceedings of a Symposium on the Neurobiology of the Amygdala, Bar Harbor, Maine, June 6–17, 1971. illustrated. New York, NY: Springer Science & Business Media.

[B45] Elharrar E. Warhaftig G. Issler O. Sztainberg Y. Dikshtein Y. Zahut R.. (2013). Overexpression of corticotropin-releasing factor receptor type 2 in the bed nucleus of stria terminalis improves posttraumatic stress disorder-like symptoms in a model of incubation of fear. Biol. Psychiatry 74, 827–836. 10.1016/j.biopsych.2013.05.039 23871471

[B46] Fendt M. Endres T. Apfelbach R. (2003). Temporary inactivation of the bed nucleus of the stria terminalis but not of the amygdala blocks freezing induced by trimethylthiazoline, a component of fox feces. J. Neurosci. 23, 23–28. 10.1523/jneurosci.23-01-00023.2003 12514197 PMC6742150

[B47] Fetterly T. L. Basu A. Nabit B. P. Awad E. Williford K. M. Centanni S. W.. (2019). α_2A_-adrenergic receptor activation decreases parabrachial nucleus excitatory drive onto BNST CRF neurons and reduces their activity *in vivo*. J. Neurosci. 39, 472–484. 10.1523/JNEUROSCI.1035-18.2018 30478032 PMC6335747

[B48] Flandreau E. I. Toth M. (2018). Animal models of PTSD: a critical review. Curr. Top. Behav. Neurosci. 38, 47–68. 10.1007/7854_2016_65 28070873

[B49] Flory J. D. Yehuda R. (2015). Comorbidity between post-traumatic stress disorder and major depressive disorder: alternative explanations and treatment considerations. Dialogues Clin. Neurosci. 17, 141–150. 26246789 10.31887/DCNS.2015.17.2/jflory PMC4518698

[B50] Forray M. I. Gysling K. (2004). Role of noradrenergic projections to the bed nucleus of the stria terminalis in the regulation of the hypothalamic-pituitary-adrenal axis. Brain Res. Rev. 47, 145–160. 10.1016/j.brainresrev.2004.07.011 15572169

[B51] Fox A. S. Oler J. A. Tromp D. P. M. Fudge J. L. Kalin N. H. (2015). Extending the amygdala in theories of threat processing. Trends Neurosci. 38, 319–329. 10.1016/j.tins.2015.03.002 25851307 PMC4417372

[B52] Garfinkel S. N. Abelson J. L. King A. P. Sripada R. K. Wang X. Gaines L. M.. (2014). Impaired contextual modulation of memories in PTSD: an fMRI and psychophysiological study of extinction retention and fear renewal. J. Neurosci. 34, 13435–13443. 10.1523/jneurosci.4287-13.2014 25274821 PMC4262698

[B53] Giardino W. J. Eban-Rothschild A. Christoffel D. J. Li S.-B. Malenka R. C. de Lecea L. (2018). Parallel circuits from the bed nuclei of stria terminalis to the lateral hypothalamus drive opposing emotional states. Nat. Neurosci. 21, 1084–1095. 10.1038/s41593-018-0198-x 30038273 PMC6095688

[B54] Giustino T. F. Maren S. (2015). The role of the medial prefrontal cortex in the conditioning and extinction of fear. Front. Behav. Neurosci. 9:298. 10.3389/fnbeh.2015.00298 26617500 PMC4637424

[B55] Gold P. W. (2015). The organization of the stress system and its dysregulation in depressive illness. Mol. Psychiatry 20, 32–47. 10.1038/mp.2014.163 25486982

[B56] Gomes-de-Souza L. Oliveira L. A. Benini R. Rodella P. Costa-Ferreira W. Crestani C. C. (2016). Involvement of endocannabinoid neurotransmission in the bed nucleus of stria terminalis in cardiovascular responses to acute restraint stress in rats. Br. J. Pharmacol. 173, 2833–2844. 10.1111/bph.13560 27441413 PMC5055136

[B57] Goode T. D. Maren S. (2017). Role of the bed nucleus of the stria terminalis in aversive learning and memory. Learn. Mem. 24, 480–491. 10.1101/lm.044206.116 28814474 PMC5580527

[B58] Goode T. D. Ressler R. L. Acca G. M. Maren S. (2018). Bed nucleus of the stria terminalis mediates fear to ambiguous threat signals. BioRxiv [Preprint]. 10.1101/376228 PMC6456295 30946011

[B59] Gourley S. L. Kedves A. T. Olausson P. Taylor J. R. (2009). A history of corticosterone exposure regulates fear extinction and cortical NR2B, GluR2/3, and BDNF. Neuropsychopharmacology 34, 707–716. 10.1038/npp.2008.123 18719621 PMC3679657

[B60] Gray S. L. Cline D. L. (2019). “PACAP,” in Stress: Physiology, Biochemistry and Pathology, ed. George Fink (San Diego, CA: Elsevier), 279–291.

[B61] Greenberg G. D. Laman-Maharg A. Campi K. L. Voigt H. Orr V. N. Schaal L.. (2014). Sex differences in stress-induced social withdrawal: role of brain derived neurotrophic factor in the bed nucleus of the stria terminalis. Front. Behav. Neurosci. 7:223. 10.3389/fnbeh.2013.00223 24409132 PMC3885825

[B62] Greenwood B. N. Foley T. E. Burhans D. Maier S. F. Fleshner M. (2005). The consequences of uncontrollable stress are sensitive to duration of prior wheel running. Brain Res. 1033, 164–178. 10.1016/j.brainres.2004.11.037 15694921

[B64] Hammack S. E. Cheung J. Rhodes K. M. Schutz K. C. Falls W. A. Braas K. M.. (2009). Chronic stress increases pituitary adenylate cyclase-activating peptide (PACAP) and brain-derived neurotrophic factor (BDNF) mRNA expression in the bed nucleus of the stria terminalis (BNST): roles for PACAP in anxiety-like behavior. Psychoneuroendocrinology 34, 833–843. 10.1016/j.psyneuen.2008.12.013 19181454 PMC2705919

[B65] Hammack S. E. Cooper M. A. Lezak K. R. (2012). Overlapping neurobiology of learned helplessness and conditioned defeat: implications for PTSD and mood disorders. Neuropharmacology 62, 565–575. 10.1016/j.neuropharm.2011.02.024 21396383 PMC3433056

[B63] Hammack S. E. May V. (2015). Pituitary adenylate cyclase activating polypeptide in stress-related disorders: data convergence from animal and human studies. Biol. Psychiatry 78, 167–177. 10.1016/j.biopsych.2014.12.003 25636177 PMC4461555

[B66] Hammack S. E. Roman C. W. Lezak K. R. Kocho-Shellenberg M. Grimmig B. Falls W. A.. (2010). Roles for pituitary adenylate cyclase-activating peptide (PACAP) expression and signaling in the bed nucleus of the stria terminalis (BNST) in mediating the behavioral consequences of chronic stress. J. Mol. Neurosci. 42, 327–340. 10.1007/s12031-010-9364-7 20405238 PMC2955825

[B67] Hammack S. E. Todd T. P. Kocho-Schellenberg M. Bouton M. E. (2015). Role of the bed nucleus of the stria terminalis in the acquisition of contextual fear at long or short context-shock intervals. Behav. Neurosci. 129, 673–678. 10.1037/bne0000088 26348716 PMC4586907

[B68] Hannibal J. Mikkelsen J. D. Clausen H. Holst J. J. Wulff B. S. Fahrenkrug J. (1995). Gene expression of pituitary adenylate cyclase activating polypeptide (PACAP) in the rat hypothalamus. Regul. Pept. 55, 133–148. 10.1016/0167-0115(94)00099-j 7754101

[B69] Hare B. D. Thornton T. M. Rincon M. Golijanin B. King S. B. Jaworski D. M.. (2018). Two weeks of variable stress increases gamma-H2AX levels in the mouse bed nucleus of the stria terminalis. Neuroscience 373, 137–144. 10.1016/j.neuroscience.2018.01.024 29352998 PMC6033054

[B70] Harris A. Seckl J. (2011). Glucocorticoids, prenatal stress and the programming of disease. Horm. Behav. 59, 279–289. 10.1016/j.yhbeh.2010.06.007 20591431

[B74] Hashimoto R. Hashimoto H. Shintani N. Ohi K. Hori H. Saitoh O.. (2010). Possible association between the pituitary adenylate cyclase-activating polypeptide (PACAP) gene and major depressive disorder. Neurosci. Lett. 468, 300–302. 10.1016/j.neulet.2009.11.019 19914336

[B71] Hashimoto H. Hashimoto R. Shintani N. Tanaka K. Yamamoto A. Hatanaka M.. (2009). Depression-like behavior in the forced swimming test in PACAP-deficient mice: amelioration by the atypical antipsychotic risperidone. J. Neurochem. 110, 595–602. 10.1111/j.1471-4159.2009.06168.x 19457081

[B72] Hashimoto H. Shintani N. Ago Y. Hayata-Takano A. Nakazawa T. Hashimoto R. (2016). “Implications of PACAP signaling in psychiatric disorders,” in Pituitary Adenylate Cyclase Activating Polypeptide — PACAP, eds Reglodi D. Tamas A. (Cham: Springer International Publishing), 757–766.

[B73] Hashimoto H. Shintani N. Tanida M. Hayata A. Hashimoto R. Baba A. (2011). PACAP is implicated in the stress axes. Curr. Pharm. Des. 17, 985–989. 10.2174/138161211795589382 21524255 PMC3179129

[B75] Hazra R. Guo J. D. Dabrowska J. Rainnie D. G. (2012). Differential distribution of serotonin receptor subtypes in BNST(ALG) neurons: modulation by unpredictable shock stress. Neuroscience 225, 9–21. 10.1016/j.neuroscience.2012.08.014 22922122 PMC3479341

[B76] Henckens M. J. A. G. Printz Y. Shamgar U. Dine J. Lebow M. Drori Y.. (2017). CRF receptor type 2 neurons in the posterior bed nucleus of the stria terminalis critically contribute to stress recovery. Mol. Psychiatry 22, 1691–1700. 10.1038/mp.2016.133 27550842

[B77] Herman J. L. (1992). Complex PTSD: a syndrome in survivors of prolonged and repeated trauma. J. Trauma. Stress 5, 377–391. 10.1007/bf00977235

[B78] Insel T. R. (2014). The NIMH research domain criteria (RDoC) project: precision medicine for psychiatry. Am. J. Psychiatry 171, 395–397. 10.1176/appi.ajp.2014.14020138 24687194

[B79] Insel T. Cuthbert B. Garvey M. Heinssen R. Pine D. S. Quinn K.. (2010). Research domain criteria (RDoC): toward a new classification framework for research on mental disorders. Am. J. Psychiatry 167, 748–751. 10.1176/appi.ajp.2010.09091379 20595427

[B80] Izquierdo A. Wellman C. L. Holmes A. (2006). Brief uncontrollable stress causes dendritic retraction in infralimbic cortex and resistance to fear extinction in mice. J. Neurosci. 26, 5733–5738. 10.1523/jneurosci.0474-06.2006 16723530 PMC6675270

[B81] Janitzky K. D’Hanis W. Kröber A. Schwegler H. (2015). TMT predator odor activated neural circuit in C57BL/6J mice indicates TMT-stress as a suitable model for uncontrollable intense stress. Brain Res. 1599, 1–8. 10.1016/j.brainres.2014.12.030 25532494

[B82] Janitzky K. Peine A. Kröber A. Yanagawa Y. Schwegler H. Roskoden T. (2014). Increased CRF mRNA expression in the sexually dimorphic BNST of male but not female GAD67 mice and TMT predator odor stress effects upon spatial memory retrieval. Behav. Brain Res. 272, 141–149. 10.1016/j.bbr.2014.06.020 24946072

[B83] Jasnow A. M. Davis M. Huhman K. L. (2004). Involvement of central amygdalar and bed nucleus of the stria terminalis corticotropin-releasing factor in behavioral responses to social defeat. Behav. Neurosci. 118, 1052–1061. 10.1037/0735-7044.118.5.1052 15506887

[B84] Jennings J. H. Sparta D. R. Stamatakis A. M. Ung R. L. Pleil K. E. Kash T. L.. (2013). Distinct extended amygdala circuits for divergent motivational states. Nature 496, 224–228. 10.1038/nature12041 23515155 PMC3778934

[B85] Jutkiewicz E. M. Wood S. K. Houshyar H. Hsin L.-W. Rice K. C. Woods J. H. (2005). The effects of CRF antagonists, antalarmin, CP154,526, LWH234 and R121919, in the forced swim test and on swim-induced increases in adrenocorticotropin in rats. Psychopharmacology 180, 215–223. 10.1007/s00213-005-2164-z 15696320 PMC1315297

[B86] Kang N. Baum M. J. Cherry J. A. (2009). A direct main olfactory bulb projection to the “vomeronasal” amygdala in female mice selectively responds to volatile pheromones from males. Eur. J. Neurosci. 29, 624–634. 10.1111/j.1460-9568.2009.06638.x 19187265 PMC2669936

[B87] Kash T. L. Pleil K. E. Marcinkiewcz C. A. Lowery-Gionta E. G. Crowley N. Mazzone C.. (2015). Neuropeptide regulation of signaling and behavior in the BNST. Mol. Cells 38, 1–13. 10.14348/molcells.2015.2261 25475545 PMC4314126

[B88] Kautz M. Charney D. S. Murrough J. W. (2017). Neuropeptide Y, resilience, and PTSD therapeutics. Neurosci. Lett. 649, 164–169. 10.1016/j.neulet.2016.11.061 27913193

[B89] Kelmendi B. Adams T. G. Yarnell S. Southwick S. Abdallah C. G. Krystal J. H. (2016). PTSD: from neurobiology to pharmacological treatments. Eur. J. Psychotraumatol. 7:31858. 10.3402/ejpt.v7.31858 27837583 PMC5106865

[B90] Kessler R. C. Avenevoli S. Costello E. J. Green J. G. Gruber M. J. Heeringa S.. (2009). Design and field procedures in the US National Comorbidity Survey Replication Adolescent Supplement (NCS-A). Int. J. Methods Psychiatr. Res. 18, 69–83. 10.1002/mpr.279 19507169 PMC2774712

[B91] Kessler R. C. Berglund P. Chiu W. T. Demler O. Heeringa S. Hiripi E.. (2004). The US national comorbidity survey replication (NCS-R): design and field procedures. Int. J. Methods Psychiatr. Res. 13, 69–92. 10.1002/mpr.167 15297905 PMC6878537

[B92] Kessler R. C. Chiu W. T. Demler O. Merikangas K. R. Walters E. E. (2005). Prevalence, severity and comorbidity of 12-month DSM-IV disorders in the National Comorbidity Survey Replication. Arch. Gen. Psychiatry 62, 617–627. 10.1001/archpsyc.62.6.617 15939839 PMC2847357

[B93] Kessler R. C. Sonnega A. Bromet E. Hughes M. Nelson C. B. (1995). Posttraumatic stress disorder in the national comorbidity survey. Arch. Gen. Psychiatry 52, 1048–1060. 10.1001/archpsyc.1995.03950240066012 7492257

[B94] Khan S. Liberzon I. (2004). Topiramate attenuates exaggerated acoustic startle in an animal model of PTSD. Psychopharmacology 172, 225–229. 10.1007/s00213-003-1634-4 14586539

[B95] King S. B. Lezak K. R. O’Reilly M. Toufexis D. J. Falls W. A. Braas K.. (2017a). The effects of prior stress on anxiety-like responding to intra-bnst pituitary adenylate cyclase activating polypeptide in male and female rats. Neuropsychopharmacology 42, 1679–1687. 10.1038/npp.2017.16 28106040 PMC5518896

[B96] King S. B. Toufexis D. J. Hammack S. E. (2017b). Pituitary adenylate cyclase activating polypeptide (PACAP), stress and sex hormones. Stress 20, 465–475. 10.1080/10253890.2017.1336535 28610473 PMC6724739

[B97] Knox D. George S. A. Fitzpatrick C. J. Rabinak C. A. Maren S. Liberzon I. (2012). Single prolonged stress disrupts retention of extinguished fear in rats. Learn. Mem. 19, 43–49. 10.1101/lm.024356.111 22240323 PMC3262971

[B98] Kocho-Schellenberg M. Lezak K. R. Harris O. M. Roelke E. Gick N. Choi I.. (2014). PACAP in the BNST produces anorexia and weight loss in male and female rats. Neuropsychopharmacology 39, 1614–1623. 10.1038/npp.2014.8 24434744 PMC4023158

[B99] Kravets J. L. Reyes B. A. S. Unterwald E. M. Van Bockstaele E. J. (2015). Direct targeting of peptidergic amygdalar neurons by noradrenergic afferents: linking stress-integrative circuitry. Brain Struct. Funct. 220, 541–558. 10.1007/s00429-013-0674-8 24271021 PMC4032379

[B100] Krystal J. H. Neumeister A. (2009). Noradrenergic and serotonergic mechanisms in the neurobiology of posttraumatic stress disorder and resilience. Brain Res. 1293, 13–23. 10.1016/j.brainres.2009.03.044 19332037 PMC2761677

[B101] Lebow M. A. Chen A. (2016). Overshadowed by the amygdala: the bed nucleus of the stria terminalis emerges as key to psychiatric disorders. Mol. Psychiatry 21, 450–463. 10.1038/mp.2016.1 26878891 PMC4804181

[B102] Lebow M. Neufeld-Cohen A. Kuperman Y. Tsoory M. Gil S. Chen A. (2012). Susceptibility to PTSD-like behavior is mediated by corticotropin-releasing factor receptor type 2 levels in the bed nucleus of the stria terminalis. J. Neurosci. 32, 6906–6916. 10.1523/jneurosci.4012-11.2012 22593059 PMC6622202

[B103] Lee Y. Davis M. (1997). Role of the hippocampus, the bed nucleus of the stria terminalis and the amygdala in the excitatory effect of corticotropin-releasing hormone on the acoustic startle reflex. J. Neurosci. 17, 6434–6446. 10.1523/jneurosci.17-16-06434.1997 9236251 PMC6568348

[B104] Lezak K. R. Missig G. Carlezon W. A. (2017). Behavioral methods to study anxiety in rodents. Dialogues Clin. Neurosci. 19, 181–191. 28867942 10.31887/DCNS.2017.19.2/wcarlezon PMC5573562

[B105] Lezak K. R. Roelke E. Harris O. M. Choi I. Edwards S. Gick N.. (2014a). Pituitary adenylate cyclase-activating polypeptide (PACAP) in the bed nucleus of the stria terminalis (BNST) increases corticosterone in male and female rats. Psychoneuroendocrinology 45, 11–20. 10.1016/j.psyneuen.2014.03.007 24845172 PMC4050443

[B106] Lezak K. R. Roman C. W. Braas K. M. Schutz K. C. Falls W. A. Schulkin J.. (2014b). Regulation of bed nucleus of the stria terminalis PACAP expression by stress and corticosterone. J. Mol. Neurosci. 54, 477–484. 10.1007/s12031-014-0269-8 24614974 PMC4162870

[B107] Liberzon I. Krstov M. Young E. A. (1997). Stress-restress: effects on ACTH and fast feedback. Psychoneuroendocrinology 22, 443–453. 10.1016/s0306-4530(97)00044-9 9364622

[B108] Lin X. Itoga C. A. Taha S. Li M. H. Chen R. Sami K.. (2018). c-Fos mapping of brain regions activated by multi-modal and electric foot shock stress. Neurobiol. Stress 8, 92–102. 10.1016/j.ynstr.2018.02.001 29560385 PMC5857493

[B109] Louvart H. Maccari S. Vaiva G. Darnaudéry M. (2009). Prenatal stress exacerbates the impact of an aversive procedure on the corticosterone response to stress in female rats. Psychoneuroendocrinology 34, 786–790. 10.1016/j.psyneuen.2008.12.002 19157714

[B110] Lupien S. J. King S. Meaney M. J. McEwen B. S. (2000). Child’s stress hormone levels correlate with mother’s socioeconomic status and depressive state. Biol. Psychiatry 48, 976–980. 10.1016/s0006-3223(00)00965-3 11082471

[B111] Maier S. F. Seligman M. E. P. (2016). Learned helplessness at fifty: insights from neuroscience. Psychol. Rev. 123, 349–367. 10.1037/rev0000033 27337390 PMC4920136

[B112] Marcinkiewcz C. A. Mazzone C. M. D’Agostino G. Halladay L. R. Hardaway J. A. DiBerto J. F.. (2016). Serotonin engages an anxiety and fear-promoting circuit in the extended amygdala. Nature 537, 97–101. 10.1038/nature19318 27556938 PMC5124365

[B113] Maren S. (2017). Synapse-specific encoding of fear memory in the amygdala. Neuron 95, 988–990. 10.1016/j.neuron.2017.08.020 28858626

[B114] Maren S. Holmes A. (2016). Stress and fear extinction. Neuropsychopharmacology 41, 58–79. 10.1038/npp.2015.180 26105142 PMC4677122

[B115] Maren S. Quirk G. J. (2004). Neuronal signalling of fear memory. Nat. Rev. Neurosci. 5, 844–852. 10.1038/nrn1535 15496862

[B116] McEwen B. S. Chattarji S. (2007). “Neuroendocrinology of stress,” in Handbook of Neurochemistry and Molecular Neurobiology, eds Lajtha A. Blaustein J. D. (Boston, MA: Springer US), 571–593.

[B300] Meaney M. J. Szyf M. (2005). Environmental programming of stress responses through DNA methylation: life at the interface between a dynamic environment and a fixed genome. Dialogues Clin. Neurosci. 7, 103–123. 10.1038/npp.2015.180 16262207 PMC3181727

[B117] Merali Z. Kent P. Du L. Hrdina P. Palkovits M. Faludi G.. (2006). Corticotropin-releasing hormone, arginine vasopressin, gastrin-releasing peptide and neuromedin B alterations in stress-relevant brain regions of suicides and control subjects. Biol. Psychiatry 59, 594–602. 10.1016/j.biopsych.2005.08.008 16197926

[B118] Merikangas K. R. He J.-P. Burstein M. Swanson S. A. Avenevoli S. Cui L.. (2010). Lifetime prevalence of mental disorders in U.S. adolescents: results from the National Comorbidity Survey Replication—Adolescent Supplement (NCS-A). J. Am. Acad. Child Adolesc. Psychiatry 49, 980–989. 10.1016/j.jaac.2010.05.017 20855043 PMC2946114

[B119] Miles O. W. Thrailkill E. A. Linden A. K. May V. Bouton M. E. Hammack S. E. (2018). Pituitary adenylate cyclase-activating peptide in the bed nucleus of the stria terminalis mediates stress-induced reinstatement of cocaine seeking in rats. Neuropsychopharmacology 43, 978–986. 10.1038/npp.2017.135 28656976 PMC5854788

[B120] Missig G. Mei L. Vizzard M. A. Braas K. M. Waschek J. A. Ressler K. J.. (2017). Parabrachial pituitary adenylate cyclase-activating polypeptide activation of amygdala endosomal extracellular signal-regulated kinase signaling regulates the emotional component of pain. Biol. Psychiatry 81, 671–682. 10.1016/j.biopsych.2016.08.025 28057459 PMC5332340

[B121] Missig G. Roman C. W. Vizzard M. A. Braas K. M. Hammack S. E. May V. (2014). Parabrachial nucleus (PBn) pituitary adenylate cyclase activating polypeptide (PACAP) signaling in the amygdala: implication for the sensory and behavioral effects of pain. Neuropharmacology 86, 38–48. 10.1016/j.neuropharm.2014.06.022 24998751 PMC4322675

[B122] Mustafa T. Jiang S. Z. Eiden A. M. Weihe E. Thistlethwaite I. Eiden L. E. (2015). Impact of PACAP and PAC1 receptor deficiency on the neurochemical and behavioral effects of acute and chronic restraint stress in male C57BL/6 mice. Stress 18, 408–418. 10.3109/10253890.2015.1025044 25853791 PMC4834918

[B123] Myers B. Mark Dolgas C. Kasckow J. Cullinan W. E. Herman J. P. (2014). Central stress-integrative circuits: forebrain glutamatergic and GABAergic projections to the dorsomedial hypothalamus, medial preoptic area and bed nucleus of the stria terminalis. Brain Struct. Funct. 219, 1287–1303. 10.1007/s00429-013-0566-y 23661182 PMC3778169

[B124] Nagaya N. Acca G. M. Maren S. (2015). Allopregnanolone in the bed nucleus of the stria terminalis modulates contextual fear in rats. Front. Behav. Neurosci. 9:205. 10.3389/fnbeh.2015.00205 26300750 PMC4523814

[B125] Nemeroff C. B. Bissette G. Akil H. Fink M. (1991). Neuropeptide concentrations in the cerebrospinal fluid of depressed patients treated with electroconvulsive therapy. Br. J. Psychiatry 158, 59–63. 10.1192/bjp.158.1.59 1673078

[B126] Newman E. L. Leonard M. Z. Arena D. T. de Almeida R. M. M. Miczek K. A. (2018). Social defeat stress and escalation of cocaine and alcohol consumption: focus on CRF. Neurobiol. Stress 9, 151–165. 10.1016/j.ynstr.2018.09.007 30450381 PMC6236516

[B127] Nievergelt C. Maihofer A. Dalvie S. Duncan L. Ratanatharathorn A. Ressler K. (2018). 157. Large-scale genetic characterization of PTSD: addressing heterogeneity across ancestry, sex, and trauma. Biol. Psychiatry 83:S64 10.1016/j.biopsych.2018.02.175

[B128] Olff M. (2017). Sex and gender differences in post-traumatic stress disorder: an update. Eur. J. Psychotraumatol. 8:1351204 10.1080/20008198.2017.1351204

[B129] Oliveira L. A. Almeida J. Benini R. Crestani C. C. (2015). CRF1 and CRF2 receptors in the bed nucleus of the stria terminalis modulate the cardiovascular responses to acute restraint stress in rats. Pharmacol. Res. 95–96, 53–62. 10.1016/j.phrs.2015.03.012 25829333

[B130] Pezuk P. Aydin E. Aksoy A. Canbeyli R. (2008). Effects of BNST lesions in female rats on forced swimming and navigational learning. Brain Res. 1228, 199–207. 10.1016/j.brainres.2008.06.071 18619949

[B131] Pham K. Nacher J. Hof P. R. McEwen B. S. (2003). Repeated restraint stress suppresses neurogenesis and induces biphasic PSA-NCAM expression in the adult rat dentate gyrus. Eur. J. Neurosci. 17, 879–886. 10.1046/j.1460-9568.2003.02513.x 12603278

[B132] Pittenger C. Duman R. S. (2008). Stress, depression, and neuroplasticity: a convergence of mechanisms. Neuropsychopharmacology 33, 88–109. 10.1038/sj.npp.1301574 17851537

[B133] Pleil K. E. Lopez A. McCall N. Jijon A. M. Bravo J. P. Kash T. L. (2012). Chronic stress alters neuropeptide Y signaling in the bed nucleus of the stria terminalis in DBA/2J but not C57BL/6J mice. Neuropharmacology 62, 1777–1786. 10.1016/j.neuropharm.2011.12.002 22182779 PMC3269561

[B134] Pleil K. E. Lowery-Gionta E. G. Crowley N. A. Li C. Marcinkiewcz C. A. Rose J. H.. (2015). Effects of chronic ethanol exposure on neuronal function in the prefrontal cortex and extended amygdala. Neuropharmacology 99, 735–749. 10.1016/j.neuropharm.2015.06.017 26188147 PMC4781662

[B135] Pomrenze M. B. Tovar-Diaz J. Blasio A. Maiya R. Giovanetti S. M. Lei K.. (2019). A corticotropin releasing factor network in the extended amygdala for anxiety. J. Neurosci. 39, 1030–1043. 10.1523/JNEUROSCI.2143-18.2018 30530860 PMC6363927

[B136] Pooley A. E. Benjamin R. C. Sreedhar S. Eagle A. L. Robison A. J. Mazei-Robison M. S.. (2018). Sex differences in the traumatic stress response: PTSD symptoms in women recapitulated in female rats. Biol. Sex Differ. 9:31. 10.1186/s13293-018-0191-9 29976248 PMC6034295

[B137] PTSD Statistics (2018). PTSD united. Available online at: http://www.ptsdunited.org/ptsd-statistics-2/. Accessed on November 19, 2018.

[B138] Qiu Z.-K. Liu C.-H. Gao Z.-W. He J.-L. Liu X. Wei Q.-L.. (2016). The inulin-type oligosaccharides extract from morinda officinalis, a traditional Chinese herb, ameliorated behavioral deficits in an animal model of post-traumatic stress disorder. Metab. Brain Dis. 31, 1143–1149. 10.1007/s11011-016-9853-7 27311612

[B139] Rainnie D. Daniel S. Menigoz A. Guo J. Ryan S. Seth S. (2017). 107. BNST cell type-selective changes in gene expression in response to chronic stress. Biol. Psychiatry 81:S45 10.1016/j.biopsych.2017.02.119

[B140] Ramikie T. S. Patel S. (2012). Endocannabinoid signaling in the amygdala: anatomy, synaptic signaling, behavior, and adaptations to stress. Neuroscience 204, 38–52. 10.1016/j.neuroscience.2011.08.037 21884761 PMC3236282

[B141] Ramikie T. S. Ressler K. J. (2016). Stress-related disorders, pituitary adenylate cyclase-activating peptide (PACAP)ergic system and sex differences. Dialogues Clin. Neurosci. 18, 403–413. 28179812 10.31887/DCNS.2016.18.4/kressler PMC5286726

[B142] Rasmusson A. M. Hauger R. L. Morgan C. A. Bremner J. D. Charney D. S. Southwick S. M. (2000). Low baseline and yohimbine-stimulated plasma neuropeptide Y (NPY) levels in combat-related PTSD. Biol. Psychiatry 47, 526–539. 10.1016/s0006-3223(99)00185-7 10715359

[B143] Regev L. Neufeld-Cohen A. Tsoory M. Kuperman Y. Getselter D. Gil S.. (2011). Prolonged and site-specific over-expression of corticotropin-releasing factor reveals differential roles for extended amygdala nuclei in emotional regulation. Mol. Psychiatry 16, 714–728. 10.1038/mp.2010.64 20548294

[B144] Reichmann F. Holzer P. (2016). Neuropeptide Y: a stressful review. Neuropeptides 55, 99–109. 10.1016/j.npep.2015.09.008 26441327 PMC4830398

[B146] Ressler R. L. Maren S. (2019). Synaptic encoding of fear memories in the amygdala. Curr. Opin. Neurobiol. 54, 54–59. 10.1016/j.conb.2018.08.012 30216780 PMC6361699

[B145] Ressler K. J. Mercer K. B. Bradley B. Jovanovic T. Mahan A. Kerley K.. (2011). Post-traumatic stress disorder is associated with PACAP and the PAC1 receptor. Nature 470, 492–497. 10.1038/nature09856 21350482 PMC3046811

[B147] Richter-Levin G. (1998). Acute and long-term behavioral correlates of underwater trauma—potential relevance to stress and post-stress syndromes. Psychiatry Res. 79, 73–83. 10.1016/s0165-1781(98)00030-4 9676829

[B148] Roman C. W. Lezak K. R. Hartsock M. J. Falls W. A. Braas K. M. Howard A. B.. (2014). PAC1 receptor antagonism in the bed nucleus of the stria terminalis (BNST) attenuates the endocrine and behavioral consequences of chronic stress. Psychoneuroendocrinology 47, 151–165. 10.1016/j.psyneuen.2014.05.014 25001965 PMC4342758

[B149] Rougemont-Bücking A. Linnman C. Zeffiro T. A. Zeidan M. A. Lebron-Milad K. Rodriguez-Romaguera J.. (2011). Altered processing of contextual information during fear extinction in PTSD: an fMRI study. CNS Neurosci. Ther. 17, 227–236. 10.1111/j.1755-5949.2010.00152.x 20406268 PMC6493793

[B150] Sah R. Ekhator N. N. Jefferson-Wilson L. Horn P. S. Geracioti T. D. (2014). Cerebrospinal fluid neuropeptide Y in combat veterans with and without posttraumatic stress disorder. Psychoneuroendocrinology 40, 277–283. 10.1016/j.psyneuen.2013.10.017 24485499 PMC4749916

[B151] Sajdyk T. J. Shekhar A. Gehlert D. R. (2004). Interactions between NPY and CRF in the amygdala to regulate emotionality. Neuropeptides 38, 225–234. 10.1016/j.npep.2004.05.006 15337374

[B152] Schmidt K. T. Makhijani V. H. Boyt K. M. Cogan E. S. Pati D. Pina M. M.. (2018). Stress-induced alterations of norepinephrine release in the bed nucleus of the stria terminalis of mice. ACS Chem. Neurosci. [Epub ahead of print]. 10.1021/acschemneuro.8b00265 30252438 PMC6500727

[B153] Schulkin J. Gold P. W. McEwen B. S. (1998). Induction of corticotropin-releasing hormone gene expression by glucocorticoids: implication for understanding the states of fear and anxiety and allostatic load. Psychoneuroendocrinology 23, 219–243. 10.1016/s0306-4530(97)00099-1 9695128

[B154] Seligman M. (1974). “Depression and learned helplessness,” in The Psychology of Depression: Contemporary Theory and Research, eds Friedman R. J. Katz M. M. (Oxford: John Wiley & Sons). Available online at: http://psycnet.apa.org/record/1975-07556-007.

[B155] Selye H. (1955). Stress and disease. Laryngoscope 65, 500–514. 10.1288/00005537-195507000-00002 13243777

[B156] Serova L. I. Laukova M. Alaluf L. G. Pucillo L. Sabban E. L. (2014). Intranasal neuropeptide Y reverses anxiety and depressive-like behavior impaired by single prolonged stress PTSD model. Eur. Neuropsychopharmacol. 24, 142–147. 10.1016/j.euroneuro.2013.11.007 24326087

[B157] Serova L. I. Tillinger A. Alaluf L. G. Laukova M. Keegan K. Sabban E. L. (2013). Single intranasal neuropeptide Y infusion attenuates development of PTSD-like symptoms to traumatic stress in rats. Neuroscience 236, 298–312. 10.1016/j.neuroscience.2013.01.040 23376740

[B158] Shalev U. Morales M. Hope B. Yap J. Shaham Y. (2001). Time-dependent changes in extinction behavior and stress-induced reinstatement of drug seeking following withdrawal from heroin in rats. Psychopharmacology 156, 98–107. 10.1007/s002130100748 11465640

[B159] Shansky R. M. (2015). Sex differences in PTSD resilience and susceptibility: challenges for animal models of fear learning. Neurobiol. Stress 1, 60–65. 10.1016/j.ynstr.2014.09.005 25729759 PMC4340080

[B160] Sherwood N. M. Krueckl S. L. McRory J. E. (2000). The origin and function of the pituitary adenylate cyclase-activating polypeptide (PACAP)/glucagon superfamily. Endocr. Rev. 21, 619–670. 10.1210/edrv.21.6.0414 11133067

[B161] Simerly R. B. Swanson L. W. (1986). The organization of neural inputs to the medial preoptic nucleus of the rat. J. Comp. Neurol. 246, 312–342. 10.1002/cne.902460304 3517086

[B162] Slominski A. T. Zmijewski M. A. Zbytek B. Tobin D. J. Theoharides T. C. Rivier J. (2013). Key role of CRF in the skin stress response system. Endocr. Rev. 34, 827–884. 10.1210/er.2012-1092 23939821 PMC3857130

[B163] Stephens M. A. C. Mahon P. B. McCaul M. E. Wand G. S. (2016). Hypothalamic-pituitary-adrenal axis response to acute psychosocial stress: effects of biological sex and circulating sex hormones. Psychoneuroendocrinology 66, 47–55. 10.1016/j.psyneuen.2015.12.021 26773400 PMC4788592

[B164] Steudte-Schmiedgen S. Stalder T. Schönfeld S. Wittchen H.-U. Trautmann S. Alexander N.. (2015). Hair cortisol concentrations and cortisol stress reactivity predict PTSD symptom increase after trauma exposure during military deployment. Psychoneuroendocrinology 59, 123–133. 10.1016/j.psyneuen.2015.05.007 26072152

[B165] Straube T. Mentzel H.-J. Miltner W. H. R. (2007). Waiting for spiders: brain activation during anticipatory anxiety in spider phobics. Neuroimage 37, 1427–1436. 10.1016/j.neuroimage.2007.06.023 17681799

[B166] Stroth N. Eiden L. E. (2010). Stress hormone synthesis in mouse hypothalamus and adrenal gland triggered by restraint is dependent on pituitary adenylate cyclase-activating polypeptide signaling. Neuroscience 165, 1025–1030. 10.1016/j.neuroscience.2009.11.023 19931358 PMC2815259

[B167] Stroth N. Holighaus Y. Ait-Ali D. Eiden L. E. (2011). PACAP: a master regulator of neuroendocrine stress circuits and the cellular stress response. Ann. N Y Acad. Sci. 1220, 49–59. 10.1111/j.1749-6632.2011.05904.x 21388403 PMC3078626

[B168] Stroth N. Kuri B. A. Mustafa T. Chan S.-A. Smith C. B. Eiden L. E. (2013). PACAP controls adrenomedullary catecholamine secretion and expression of catecholamine biosynthetic enzymes at high splanchnic nerve firing rates characteristic of stress transduction in male mice. Endocrinology 154, 330–339. 10.1210/en.2012-1829 23221599 PMC3529367

[B169] Sullivan G. M. Apergis J. Bush D. E. A. Johnson L. R. Hou M. Ledoux J. E. (2004). Lesions in the bed nucleus of the stria terminalis disrupt corticosterone and freezing responses elicited by a contextual but not by a specific cue-conditioned fear stimulus. Neuroscience 128, 7–14. 10.1016/j.neuroscience.2004.06.015 15450349

[B170] Timpl P. Spanagel R. Sillaber I. Kresse A. Reul J. M. Stalla G. K.. (1998). Impaired stress response and reduced anxiety in mice lacking a functional corticotropin-releasing hormone receptor 1. Nat. Genet. 19, 162–166. 10.1038/520 9620773

[B171] Todd T. P. Vurbic D. Bouton M. E. (2014). Behavioral and neurobiological mechanisms of extinction in Pavlovian and instrumental learning. Neurobiol. Learn. Mem. 108, 52–64. 10.1016/j.nlm.2013.08.012 23999219 PMC3946264

[B172] Torrisi S. Gorka A. X. Gonzalez-Castillo J. O’Connell K. Balderston N. Grillon C.. (2018). Extended amygdala connectivity changes during sustained shock anticipation. Transl. Psychiatry 8:33. 10.1038/s41398-017-0074-6 29382815 PMC5802685

[B173] Trainor B. C. Pride M. C. Villalon Landeros R. Knoblauch N. W. Takahashi E. Y. Silva A. L.. (2011). Sex differences in social interaction behavior following social defeat stress in the monogamous California mouse (*Peromyscus californicus*). PLoS One 6:e17405. 10.1371/journal.pone.0017405 21364768 PMC3045459

[B174] Trask S. Bouton M. E. (2018). Retrieval practice after multiple context changes, but not long retention intervals, reduces the impact of a final context change on instrumental behavior. Learn. Behav. 46, 213–221. 10.3758/s13420-017-0304-z 29234996 PMC5970024

[B175] Trask S. Thrailkill E. A. Bouton M. E. (2017). Occasion setting, inhibition, and the contextual control of extinction in Pavlovian and instrumental (operant) learning. Behav. Processes 137, 64–72. 10.1016/j.beproc.2016.10.003 27720958 PMC5768202

[B176] Tsigos C. Chrousos G. P. (2002). Hypothalamic-pituitary-adrenal axis, neuroendocrine factors and stress. J. Psychosom. Res. 53, 865–871. 10.1016/s0022-3999(02)00429-4 12377295

[B177] Tsukiyama N. Saida Y. Kakuda M. Shintani N. Hayata A. Morita Y.. (2011). PACAP centrally mediates emotional stress-induced corticosterone responses in mice. Stress 14, 368–375. 10.3109/10253890.2010.544345 21438773 PMC3128825

[B178] Vale S. (2005). Psychosocial stress and cardiovascular diseases. Postgrad. Med. J. 81, 429–435. 10.1136/pgmj.2004.028977 15998817 PMC1743305

[B179] Vaudry D. Hamelink C. Damadzic R. Eskay R. L. Gonzalez B. Eiden L. E. (2005). Endogenous PACAP acts as a stress response peptide to protect cerebellar neurons from ethanol or oxidative insult. Peptides 26, 2518–2524. 10.1016/j.peptides.2005.05.015 16009465 PMC4183202

[B180] Vyas A. Bernal S. Chattarji S. (2003). Effects of chronic stress on dendritic arborization in the central and extended amygdala. Brain Res. 965, 290–294. 10.1016/s0006-8993(02)04162-8 12591150

[B181] Waddell J. Morris R. W. Bouton M. E. (2006). Effects of bed nucleus of the stria terminalis lesions on conditioned anxiety: aversive conditioning with long-duration conditional stimuli and reinstatement of extinguished fear. Behav. Neurosci. 120, 324–336. 10.1037/0735-7044.120.2.324 16719697

[B182] Walker D. L. Davis M. (1997). Double dissociation between the involvement of the bed nucleus of the stria terminalis and the central nucleus of the amygdala in startle increases produced by conditioned versus unconditioned fear. J. Neurosci. 17, 9375–9383. 10.1523/jneurosci.17-23-09375.1997 9364083 PMC6573581

[B183] Walker D. L. Miles L. A. Davis M. (2009). Selective participation of the bed nucleus of the stria terminalis and CRF in sustained anxiety-like versus phasic fear-like responses. Prog. Neuropsychopharmacol. Biol. Psychiatry 33, 1291–1308. 10.1016/j.pnpbp.2009.06.022 19595731 PMC2783512

[B184] Walker D. L. Toufexis D. J. Davis M. (2003). Role of the bed nucleus of the stria terminalis versus the amygdala in fear, stress, and anxiety. Eur. J. Pharmacol. 463, 199–216. 10.1016/s0014-2999(03)01282-2 12600711

[B185] Ward H. E. Johnson E. A. Salm A. K. Birkle D. L. (2000). Effects of prenatal stress on defensive withdrawal behavior and corticotropin releasing factor systems in rat brain. Physiol. Behav. 70, 359–366. 10.1016/s0031-9384(00)00270-5 11006435

[B186] Yehuda R. (2002). Post-traumatic stress disorder. N. Engl. J. Med. 346, 108–114. 10.1056/NEJMra012941 11784878

[B187] Yehuda R. Engel S. M. Brand S. R. Seckl J. Marcus S. M. Berkowitz G. S. (2005). Transgenerational effects of posttraumatic stress disorder in babies of mothers exposed to the World Trade Center attacks during pregnancy. J. Clin. Endocrinol. Metab. 90, 4115–4118. 10.1210/jc.2005-0550 15870120

[B188] Zimmerman J. M. Maren S. (2011). The bed nucleus of the stria terminalis is required for the expression of contextual but not auditory freezing in rats with basolateral amygdala lesions. Neurobiol. Learn. Mem. 95, 199–205. 10.1016/j.nlm.2010.11.002 21073972 PMC3050017

[B189] Zorrilla E. P. Koob G. F. (2010). Progress in corticotropin-releasing factor-1 antagonist development. Drug Discov. Today 15, 371–383. 10.1016/j.drudis.2010.02.011 20206287 PMC2864802

